# A comprehensive roadmap for MedTech innovations uptake into the public healthcare system in India

**DOI:** 10.3389/fdgth.2023.1268010

**Published:** 2023-12-01

**Authors:** Prakamya Gupta, Charu Rai, Anjaney Shahi, Manisha Sharma, Ranjan Choudhury, Atul Kotwal

**Affiliations:** Healthcare Technology Division, National Health Systems Resource Center (NHSRC), Munirka, India

**Keywords:** low and middle income countries, medical devices, medical technology (Med-Tech) innovations, healthcare innovation, public health

## Abstract

**Background:**

The burden of communicable, non-communicable diseases and reproductive maternal, newborn, child & adolescent health in India, reflects the necessity to develop tailored solutions. The plethora of MedTech innovations has provided healthcare facilities with more effective, affordable and accessible healthcare for people across the country. However, in spite of the *Make-in-India* scheme in the country, the indigenously developed healthcare technology is far from making an impact on the healthcare system.

**Objective:**

To present a roadmap for MedTech innovations for their successful deployment into the public healthcare system.

**Methodology:**

In addition to the literature review, recommendations were included from several stakeholders such as innovators, manufacturers, policymakers, subject matter experts, funding organizations, State health officials etc.

**Results and conclusion:**

The journey of healthcare innovation from need identification to ideation, to prototyping and validation has paved the way towards the *de novo* design that caters to unmet needs. Innovations at the advanced technology readiness level (TRL 7/8 and above) demand a holistic and multidisciplinary approach which includes clinical validation, regulatory approval and Health technology assessment. The deployment of healthcare technology into the public healthcare system must consider resources (e.g., time, staff, budget, investment policies), ethical concerns (privacy, security, regulations, ownership), governance (policy, accountability, responsibility etc.), and Skills (capabilities, culture, etc.). The technologies are considered for field trials before the uptake in the public health system. Technology can be a key tool in achieving Universal Health Coverage but its use has to be strategic, judicious, and cognizant of issues around privacy and patient rights.

## Introduction

**Figure 1:**
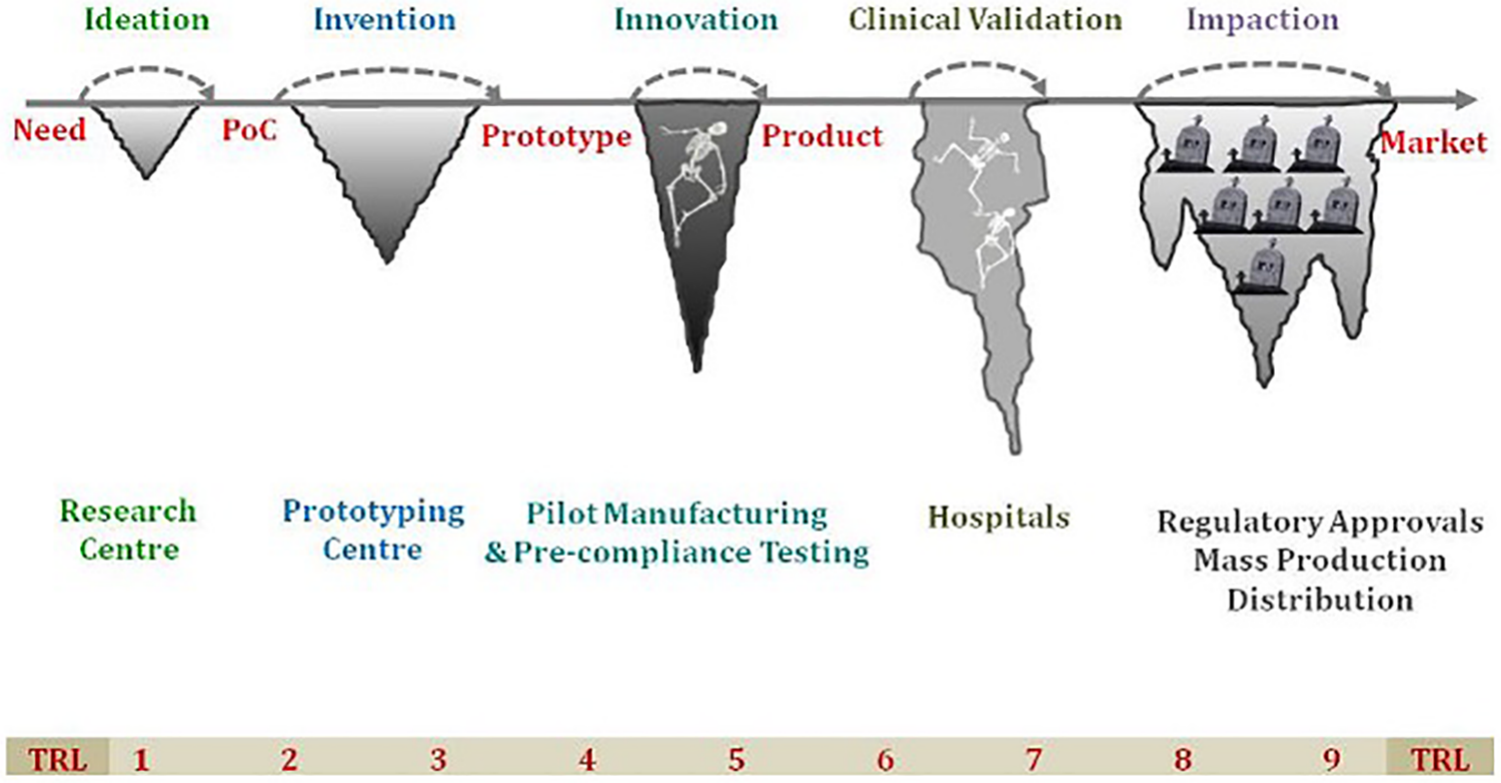
The “Valley of Death” highlighting the various challenges faced in transition from ideation to successful commercialization of the product. TRL, technology readiness level; PoC, point-of-care.

Recently, “Innovation” has become a buzzword in the MedTech sector. Modern technologies such as mHealth, wearable devices, point-of-care *in vitro* diagnostics and medical devices have made a significant impact on Non-Communicable Diseases (NCD) leading to improved health outcomes by early diagnosis and reduced morbidity and mortality. The technology has also resulted in improved DALYs and QALYs by using point of care diagnostics, IT based platforms and other screening strategies. To tackle the country's health needs and reach the marginalized population in remote areas, healthcare technologies have become a huge enabler in ensuring the delivery of quality and affordable healthcare. The interest in medical devices and diagnostic products is on the rise and there are several notable start-ups and small and medium enterprises working on innovative solutions or already made an impact in the Indian context. The technologies developed in the developed countries may not provide a tailored solution to the low and middle income countries (LMICs) due to varied geographies and local conditions. This has led to the development of a plethora of indigenous Med-Tech innovations to provide more effective, more affordable, and more accessible healthcare across the country. However, there is a mixed bag of obstacles in the transition from ideation to getting regulatory approval to commercialization which is well known and often referred to as “*Valleys of Death”* (Figure 1). The medical device policy and other recent Government of India initiatives such as “Self-reliant India” (Atmanirbhar Bharat), “Make-in-India” and “Digital India” have further promoted the med-tech sector in the country (1). Despite these efforts, indigenous innovations are struggling to leapfrog the hurdles and significantly impact the public health system.

Innovation is a broad concept which encompasses three types of innovations: (i) healthcare products which includes devices or diagnostic products (ii) healthcare services and (iii) software/mobile apps. Technology Readiness Level (TRL) is a common scale for grading innovation depending on what part of the development process is going to be ranked (2). The standard course for MedTech innovation is to be superseded by more sophisticated, advanced and user-friendly technology. This article delineates a roadmap for advanced-stage (TRL 7+) healthcare innovation and suggests how to overcome the barriers and use the technology for wider societal benefit.

## Clinical validation of Med-Tech innovation

Unlike well-established drugs and vaccine trials, medical devices or diagnostic product trials are very different from drug trials. There exists a gap in standard validation protocols for medical devices and diagnostic products in the country. Device trials tend to be cumbersome with diverse endpoints and usually depend on the physician technique. Unlike drug trials, device trials are difficult to blind, randomize or control (3). It is imperative to cover device validation and revalidation throughout the product lifecycle, from design through post-market. Typically, a single pivotal trial follows the feasibility stage(s). The device trials are designed to support a “reasonable assurance of safety and effectiveness” for the marketing application. The term, “Gold standard” is considered as an available measurement per consensus, against which the accuracy of other measurements of similar purposes may be judged (4). The device trial needs comparison to either a Gold standard or existing technology or health outcome or baseline data. The devices or diagnostics under investigation are often compared to the available so called “Gold standard” for their sensitivity and specificity. For novel medical devices, finding the gold standard is extremely difficult as technology is bound to change at a fast pace and forms the basis of innovation to newly improved standards.

## Medical device parks for scale-up of technologies

There are large numbers of med-tech innovations that have the potential to translate to impactful healthcare solutions. However, the infrastructure and resources to scale-up individually become a limiting factor. With limited resources, it would be useful to cluster infrastructure and knowledge requirements as a centralized resource. The common facility centers provide necessary infrastructure and support for innovators to scale-up the product. Currently, several Medical Device Parks [(i) Andhra Pradesh Med-tech Zone Ltd. (AMTZ), Andhra Pradesh, (ii) Telangana Medical Device Park, Telangana (iii) Kerala State Industrial Development Corporation (KSIDC), Kerala & (iv) HLL, Medipark Ltd. (HML), Tamil Nadu] are already established in the Country and many more are in the pipeline.

## Regulatory approvals

The Central Drug Standard Control Organization (CDSCO) acts as a national regulatory authority under the Drug Controller General of India (DCGI), Ministry of Health and Family Welfare, Government of India and is responsible for laying down the standards and ensuring safety, efficacy & quality for Medical Devices. The CDSCO provides approval for manufacturing the product in the country. CDSCO's State Licencing Authority (SLA) or Central Licencing Authority (CLA) grants approvals. The CDSCO published new medical device and IVD regulations to replace the country's Drugs and Cosmetics Act in 2017. The rules aim to provide a conducive environment for fostering India-specific innovation and improve accessibility and affordability of medical devices across the globe by leveraging the comparative cost advantage of manufacturing in India. The new rules establish a risk-based approach, whereby the level of regulation varies considerably depending on the risks and technology associated with the device's intended use and technological characteristics. Four classes (A, B, C and D) have been established under the new framework, where class A and B present the least risk and class C and D devices present a higher risk to patients (5). Based on this classification, the degree of validation strategy must satisfy the criteria of acceptability, repeatability and validity, besides others such as simplicity, safety, rapidity, ease of administration and cost. The product most likely to fulfill one condition may however, be least likely to fulfill another for e.g., a product with greater accuracy may be more expensive and time-consuming. The innovator/ manufacturer can voluntarily obtain a quality certification scheme for medical devices- Indian Certification of Medical Devices (ICMED). These are based on the international quality management system standards with additional requirements which are specific to India's requirements. (i) ICMED 13485—based on the International Harmonized Standard (ISO 13485) “Quality Management Systems for Medical Devices” plus additional requirements specified under the scheme. (ii) ICMED 9000—based on the International Harmonized Standard (ISO 9000:2008), “Quality Management Systems.”

## Health technology assessment

With the advent of disruptive technologies like artificial intelligence and machine learning, medical device development has taken new heights. This has given rise to evidence-based innovations that focus on usefulness, safety, affordability and acceptability in the Indian market scenario in order to maximize the benefits that can be gained with a limited healthcare budget. Health technology assessment (HTA) is a multidirectional approach that focuses on cost and clinical effectiveness, policy and ethical perspectives to provide evidence that forms the basis for rational decision-making, for the deployment of health technologies across the country. The HTA offers an evidence-based approach for assessment, evaluation and use of medical devices or diagnostic products in public health facilities in India. Establishing the HTA tool for robust policy-making will ensure the effective deployment of affordable and accessible and cost-effective technologies to states, in order to realize the ambitious dream of the national health mission (6–8). However, limited capacities for undertaking HTA impede expeditious technology evaluation and their procurement through government channels. The HTA India (HTAIn) under the Department of Health Research (DHR), Ministry of Health and Family Welfare is the nodal agency for conducting HTA in the country. HTAIn is comprised of five core bodies—the Secretariat, the Technical Appraisal Committee (TAC), Regional Resource Hubs (RRH), Technical Partners (TPs), and the HTAIn Board.

## Startup incubator

The government's startup initiatives prioritize incubators by providing them with recognition, regulatory standards, and financial assistance. There are presently over 250 recognised incubators in the nation (including the 56 under the startup policy), with some of them sponsored by prestigious educational institutions such as IIMs and IITs. These incubators provide innovation ecosystem support comprizing of technology facilities, guidance, seed funds, network and connections, co-working spaces, lab facilities, mentorship, and advisory services.

## State innovation hubs (SIH)

There is a felt need to institutionalize a structured mechanism within existing NHM to recognize relevant problems that enable the development of evidence-based sustainable solutions and moving away from a one-time experiment with limited funds for testing innovative ideas. Some of these ideas and interventions provide appreciable outcomes but are not carried forward due to limited resources. An institutionalized mechanism will effectively address public health priorities and lead to the integration of approved innovative ideas in the state implementation system in a sustainable manner under the National Health Mission. The proposed State Innovation Hub (SIH) under NHM is an institutional mechanism to initiate a sustained process for technology identification. SIH also helps increase evidence for the provision and improvement of public healthcare services and recognizes areas of concern and works on them through evidence-based new or innovative interventions. These innovation Hubs drive the development process of innovations and provide the appropriate platform and ecosystem to test selected innovations, in pilot mode, which holds the potential to create sustainable solutions to address the public health needs of the state.

## Deployment of innovations into the public health system

The Government of India has always encouraged piloting and scaling up innovations and good practices for improved health outcomes. There is a need to systematically identify innovations and good practices in the country which can have a high impact to address morbidity & mortality and facilitate their prompt uptake and scale up are encouraged through a platform providing repository, learnings, and cross-learning. Recently, state innovation hubs within the State Health Resource Centers have also been encouraged through the Program Implementation Plan (PIP) route under NHM.

The National Healthcare Innovation Portal (https://nhinp.org) developed by the Ministry of Health & Family Welfare, Govt of India serves as a platform to receive innovations in various categories like good replicable practices & health product innovations. The health product innovations with technology readiness level (TRL) 7+ onwards are screened based on the inclusion/exclusion criteria (Table 1; Supplementary Table S1). The shortlisted health innovation products after HTA are recommended for disposal as follows:

a. Piloting an Innovation: In case validation data is not sufficient then the product could be recommended for further clinical validation study and in order to evaluate the feasibility of the product in the public healthcare system. For conducting a pilot, it is pertinent to identify the study sites, key performance indicators, implementation design, monitoring and evaluation checklist and cost-effectiveness analysis. Accordingly, the states can propose a budget for approved innovations in Program Implementation Plan (PIP) for pilot studies. The feasibility pilots in a few selected districts incorporate both patients' and healthcare providers' perspectives. Building trust is very important for the adoption of any new innovative technology. The trust is built upon the quality and reliability factors of the intervention and acceptance is easily made when the innovation is fulfilling the gap in the system leading to improved health outcomes.
b. Uptake in the public health system: Technologies tested in the field and recognised as enablers for improved health outcomes are showcased in innovations summits and recommended to States/UTs for uptake under various health programs.

**Table 1:** Model healthcare product innovation assessment criteria.

Scoring sheet for MedTech innovation				
Innovation title:				
S.No.	Questions	Criteria	Max score (Yes/No)	Score Obtained
1	What is technology's stage of development	Fully commercialized and listed on GeM (TRL 9)	3	
		Fully commercialized but not listed on GeM (TRL9)	2	
		Pre commercialized (TRL 8 & below)	1	
2	Does the technology target a well-defined and substantial health- problem		0–3	
3	Is the innovation novel, unique and commercially viable?	Novel & unique technology	1	
		Patent obtained	1	
4	Superiority of technology in terms of safety and efficiency?	Regulatory approval obtained (CDSCO)	2	
		Cost effective in public healthcare setting	1	
5	Will the product lead to positive health outcomes in low resource settings?		0–2	
6	Is it a well-balanced, committed and resourceful team?	Innovation backed by Govt org. (ICMR/DBT/DST/BIRAC/IITs)	1	
		Clear, concise and professional presentation	1	
Total Score*			15	
Grading of Innovation	14–15	Recommended for pilot/uptake in public health programs		
	10–13	Recommended for Health Technology Assessment (HTA)		
	0–9	Not Recommended		

TRL, technology readiness level; GeM, Government eMarketplace.

The State Program Implementation Plan (PIPs) spell out the strategies to be deployed, budgetary requirements and health outcomes aimed for. The Program Implementation Plan (PIP) is an annual process of planning, approval and allocation of budgets of various programmes under the National Health Mission (NHM) for all the States/UTs. The States are encouraged to pilot and uptake new innovative technologies and submit proposals under the category Innovations—State Specific Programme Innovations and Interventions. The Government of India appraises the proposals received from the States/UTs and approvals/support is provided based on the discussion held during the National Program Coordination Committee (NPCC) meetings.

## Conclusion

Indigenously developed novel healthcare technologies have the potential to transform the healthcare system and reduce healthcare disparity (9). The burden of diseases, ethnicity and cost of devices varies from country to country. In India, the healthcare and medical device industries have risen significantly during the previous decade. India is Asia's fourth largest market for medical equipment and it is expected to grow at a CAGR of 15% whereas the diagnostic market is likely to expand at a CAGR of 13.5% (10). Government initiatives such as “Self-reliance India” (Atmanirbhar Bharat), “Make-in-India,” Start-up India and “Digital India” have boosted the country's med-tech sector.

Several new technologies such as point-of-care diagnostics, Artificial Intelligence in Healthcare, Blockchain management, telemedicine, wearable technology & health monitoring are under integration into the public health system. It is imperative to incentivize and motivate local manufacturing in India and provide holistic support to the technologies/ products. The academia-Industry linkage may pave the way for boosting research and development and promoting clinical validation of medical devices and diagnostics. However, guidelines are required to streamline Intellectual Property (IP) sharing, revenue sharing, faculty/institute role in the IP and other issues between the academic institutes and industrial partners.

From need identification to ideation, validation and prototyping, Biodesign programs have a unique approach that paved the way towards the predictable *de novo* design and development of med-tech innovation. A multi-disciplinary team comprising doctors, engineers and design experts is vital in making this a success. The involvement of clinicians, especially from medical colleges is critical for successful Med-tech innovations.

With these insights, India is striding towards a self-reliant nation and working to leverage technology to achieve Universal Health Coverage.

## Data Availability

The original contributions presented in the study are included in the article/**Supplementary Material**, further inquiries can be directed to the corresponding authors.
