# Supplementary material for: A comprehensive roadmap for MedTech innovations uptake into the public healthcare system in India
Source: Front Digit Health. 2023 Dec 1;5:1268010. doi: 10.3389/fdgth.2023.1268010 (PMC10722438; doi:10.3389/fdgth.2023.1268010)
Supplement: Supplementary file 1 [file Table1.docx]

**Supplementary Table 1:**  Model Healthcare Technologies Assessment Form

| **S.No.** | **Technology Domain** | **Questions** | **Remarks** |
| --- | --- | --- | --- |
| 1 | Innovator Details | Name of Innovator |  |
|  |  | Company Name & Address |  |
|  |  | Mobile No: |  |
|  |  | Email Id: |  |
| 2 | Technology Details | Name of Technology |  |
|  |  | Technology Readiness Level (TRL) |  |
|  |  | CDSCO/ DCGI Certification | Yes/ No |
|  |  | Technology classification as per Medical Device Rules 2017 (Class A, B, C,D) |  |
|  |  | BIS Certified | Yes/ No |
|  |  | Any other certification |  |
| 3 | Technology Validation | Whether the technology validated? | Yes/ No |
|  |  | Third party validation | Yes/ No |
|  |  | Govt / PSU/ Institute where the technology was validated |  |
|  |  | Validation protocol |  |
|  |  | Validation Results |  |
|  |  | Efficacy/ Efficiency or Sensitivity or Specificity |  |
| 4 | Target population | Does the technology targets any particular disease/ national program |  |
|  |  | Is the technology specific for a particular region/ community |  |
|  |  | Intervention level (Select one) | Preventive  Curative  Promotive  Rehabilitative |
|  |  | Entire population | Yes/ No |
| 5 | Novelty | Patented Technology/ Patent Filed |  |
|  |  | Is this *Make-In-India* Product/ technology | Yes/ No |
|  |  | % of indigenous component | <25 %  25-50 %  50-75 %  >75-99 %  100% |
| 6 | Technology outcome | Is the technology ready for uptake at all levels of healthcare settings. | Yes/ No |
|  |  | Is the technology effective in primary and secondary Setting | Yes/ No |
|  |  | Additional resources required for working at Healthcare setting |  |
|  |  | Training required for operationalisation of the technology |  |
|  |  | Does the technology compromises patient safety |  |
|  |  | Frequency of Maintenance required |  |
|  |  | Availability of Maintenance |  |
| 7 | Scalability | Large-scale manufacturing capacity (Yes/No) |  |
| 8 | Cost | Per unit Cost | Mention Value |
| 9 | Reference papers/ website | |  |

BIS: Bureau of Indian Standards; CDSCO: Central Drugs Standard Control Organisation; TRL: Technology readiness level
